# On the role of ozone feedback in the ENSO amplitude response under global warming

**DOI:** 10.1002/2016GL072418

**Published:** 2017-04-30

**Authors:** Peer J. Nowack, Peter Braesicke, N. Luke Abraham, John A. Pyle

**Affiliations:** ^1^ Department of Chemistry, Centre for Atmospheric Science University of Cambridge Cambridge UK; ^2^ IMK‐ASF Karlsruhe Institute of Technology Karlsruhe Germany; ^3^ National Centre for Atmospheric Science UK

**Keywords:** ozone, climate change, ENSO, Walker circulation, global warming, climate variability

## Abstract

The El Niño–Southern Oscillation (ENSO) in the tropical Pacific Ocean is of key importance to global climate and weather. However, state‐of‐the‐art climate models still disagree on the ENSO's response under climate change. The potential role of atmospheric ozone changes in this context has not been explored before. Here we show that differences between typical model representations of ozone can have a first‐order impact on ENSO amplitude projections in climate sensitivity simulations. The vertical temperature gradient of the tropical middle‐to‐upper troposphere adjusts to ozone changes in the upper troposphere and lower stratosphere, modifying the Walker circulation and consequently tropical Pacific surface temperature gradients. We show that neglecting ozone changes thus results in a significant increase in the number of extreme ENSO events in our model. Climate modeling studies of the ENSO often neglect changes in ozone. We therefore highlight the need to understand better the coupling between ozone, the tropospheric circulation, and climate variability.

## Key Points

- The representation of ozone in climate change simulations can lead to major differences in projected ENSO amplitudes
- Mechanistically, this effect is directly related to an ozone‐induced damping of the Walker circulation response to CO_2_ forcing
- ENSO modeling studies with climatologically inconsistent ozone might be missing an important mechanism

## 1 Introduction

The El Niño–Southern Oscillation (ENSO) is an atmosphere‐ocean coupled climate mode in the tropical Pacific with an irregular period of ~2–7 years [*McPhaden et al*., 2006; *Guilyardi et al*., 2009]. It is characterized by positive or negative sea surface temperature (SST) anomalies in the East Pacific and Central Pacific. Events with positive SST anomalies are referred to as El Niño and events with negative anomalies as La Niña. The ENSO is of great relevance for short‐term climate variability and extreme weather events worldwide due to the dynamical coupling of the tropical Pacific to remote regions via atmospheric teleconnections [*Bjerknes*, 1969; *Christensen et al*., 2013; *Allen et al*., 2015]. ENSO‐induced weather extremes have been linked to severe economic and human health impacts in countries around the world [*Kovats et al*., 2003; *McPhaden et al*., 2006; *Vecchi and Wittenberg*, 2010].

Atmospheric ozone is a greenhouse gas and major absorber of solar radiation. Ozone's distribution is predicted to change under greenhouse gas forcing. So far, only impacts of different ENSO states on global tropospheric and stratospheric ozone concentrations have been explored [e.g., *Zeng and Pyle*, 2005; *Lin et al*., 2014, 2015; *Neu et al*., 2014]. Here we demonstrate for the first time the potential of ozone changes to alter the response of the tropical Walker circulation and the ENSO to increased atmospheric carbon dioxide (CO_2_) concentrations.

## 2 Methods

### 2.1 The Model

We use the Hadley Centre Global Environment Model version 3 (HadGEM3‐AO) from the United Kingdom Met Office [*Hewitt et al*., 2011]. The atmosphere is represented by the Met Office's Unified Model (MetUM) version 7.3 using a regular grid with a horizontal resolution of 3.75° longitude by 2.5° latitude and 60 vertical levels up to a height of ~84 km. The ocean component is the Océan Parallélisé part of the Nucleus for European Modelling of the Ocean (NEMO) model version 3.0 [*Madec*, 2008] coupled to the Los Alamos sea ice model CICE version 4.0 [*Hunke and Lipscomb*, 2008]. The NEMO configuration used here deploys a tripolar, locally anisotropic grid which has 2° resolution in longitude everywhere but an increased latitudinal resolution in certain regions with up to 0.5° in the tropics. Within 31 levels, NEMO reaches down to a depth of 5 km.

Atmospheric chemistry is represented by the United Kingdom Chemistry and Aerosols (UKCA) model in an updated version of the detailed stratospheric chemistry configuration [*Morgenstern et al*., 2009; *Nowack et al*., 2015, 2016] which is coupled to the MetUM. A relatively simple tropospheric chemistry scheme that simulates hydrocarbon oxidation is included. Photolysis rates are calculated interactively using the Fast‐JX photolysis scheme [*Telford et al*., 2013]. In total 159 chemical reactions involving 41 chemical species are considered.

### 2.2 The Simulations

In order to study the impact of different model representations of ozone on ENSO projections, we first carried out a range of preindustrial control simulations (piControl, 285 ppmv CO_2_) and, second, typical climate sensitivity simulations in which atmospheric CO_2_ was abruptly quadrupled to four times its preindustrial value (hereafter referred to as 4xCO_2_, 1140 ppmv CO_2_). These simulations are standard experiments in model intercomparison projects [*Taylor et al*., 2012; *Kravitz et al*., 2013]. We simulated both the low and the high CO_2_ climate using different representations of ozone in the model (see overview in Table 1).

**Table 1:** Type of Simulation, Run Label, Representation of Ozone, NINO3.4, and NINO3 Standard Deviations *σ* of SST Anomalies in These Regions (Often Referred to as ENSO Amplitudes)^a^

Type	Label	Representation of Ozone	*σ*_NINO3.4_ (K)	*σ*_NINO3_ (K)
piControl	A	*Interactive*	0.81	0.74
piControl	A1	Climatology from A	−0.02	+0.02
piControl	A2	Climatology from A	+0.06	+0.06
4xCO_2_	B	*Interactive*	+0.08	+0.12
4xCO_2_	C1	Climatology from A	+0.37	+0.43
4xCO_2_	C2	Climatology from A	+0.37	+0.39
4xCO_2_	D1	*Troposphere* + *3 levels* then climatology from A	+0.24	+0.27
4xCO_2_	D2	*Troposphere* + *3 levels* then climatology from A	+0.21	+0.22

a Absolute values are shown for experiment A and differences relative to A for all other experiments. *σ* is calculated from the last 150 years of each 200 year long 4xCO_2_ run and 150 years of each piControl run. The data were linearly detrended after calculating 5 months running means. Italics highlight where chemistry is interactive.

To set benchmarks, we ran both the piControl and 4xCO_2_ experiment in a model configuration in which HadGEM3‐AO is fully coupled to UKCA. The calculated ozone distributions are thus internally consistent with the actual state of the atmosphere simulated by HadGEM3‐AO (ozone production and depletion depend on many variables such as the solar flux, pressure, temperature, and abundances of other chemical species in each model grid cell). The changes in ozone (also “ozone feedbacks”), in turn, were then allowed to feed back onto the climate system. For example, ozone absorbs solar and terrestrial radiation and thus impacts atmospheric temperatures. These runs with interactive chemistry are referred to as “A” (piControl) and “B” (4xCO_2_).

In addition to these “interactive” runs, we carried out simulations in which HadGEM3‐AO was not coupled to UKCA. In these “noninteractive” cases, ozone and other major radiatively active trace gas species (methane and nitrous oxide) are imposed as fixed climatologies that cover both the seasonal cycle and the model's spatial dimensions. The use of ozone climatologies of this kind in climate models without interactive chemistry is commonplace [e.g., *Son et al*., 2008; *Cionni et al*., 2011; *Jones et al*., 2011; *Kravitz et al*., 2013; *Nowack et al*., 2015]. We performed two versions of each noninteractive experiment (labels 1 and 2), where the climatologies were zonally averaged in runs with label 2 (see supporting information for details). The results for each pair of integrations are almost identical so that we consider them as two ensemble members.

In the 4xCO_2_ simulations C1/C2, we used preindustrial ozone climatologies derived as time average from piControl run A. Consequently, changes in ozone mass mixing ratios, or ozone feedbacks, in response to the 4xCO_2_ forcing are not considered. In the 4xCO_2_ simulations D1/D2, we emulated the model setup applied by the UK Met Office in the abrupt 4xCO_2_ simulation carried out with the HadGEM2‐ES climate model for the Coupled Model Intercomparison Project Phase 5 [*Jones et al*., 2011]. There, interactive ozone chemistry was included below the tropopause [*Hoerling et al*., 1993] and three model levels above (equaling ~3–4 km of the stratosphere). At higher altitudes, a preindustrial ozone climatology was imposed, just as in C1/C2.

All 4xCO_2_ simulations (i.e., B, C1/C2, and D1/D2) were run for 200 years after the abrupt 4xCO_2_ forcing. For analysis, the last 150 years of each 4xCO_2_ simulation are compared to 150 years of each piControl run (where A1/A2 are the noninteractive alternatives to A). By design, any significant climatological differences among the 4xCO_2_ simulations must be caused by variations of the representation of ozone in the model.

Previously, we have shown that the use of preindustrial ozone in C1/C2 leads to significantly greater global warming in response to the 4xCO_2_ forcing than for the interactive run B [*Nowack et al*., 2015]. Tropical upper tropospheric and lower stratospheric ozone changes were identified as a key driver behind the smaller global warming in B. Other models found a similar mechanism with somewhat smaller global mean impact [e.g., *Dietmüller et al*., 2014].

## 3 Results

### 3.1 ENSO Amplitude Changes

**Figure 1:**
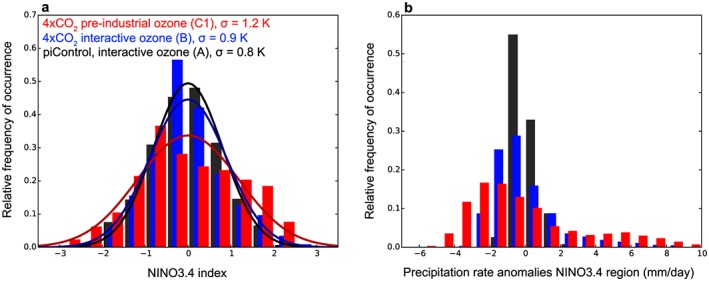
Normalized histograms of NINO3.4 (a) temperature and (b) precipitation rate anomalies as relative frequencies of occurrence, color coding for the integrations as in Figure 1a.

ENSO amplitudes are typically measured statistically as standard deviations *σ* of SST anomalies in a range of defined ENSO index regions [*Bellenger et al*., 2014; *Cai et al*., 2015a]. Figure 1a shows histograms of SST anomalies within the NINO3.4 region (170W–120W, 5N–5S) for simulations A, B, and C1, superimposed by their zero‐centered normal distributions. The figure highlights significant differences in ENSO amplitudes (i.e., *σ*) and more generally SST anomaly distributions between these simulations (the statistical robustness is discussed in detail in the supporting information). The NINO3.4 amplitude (*σ*_NINO3.4_) under 4xCO_2_ with interactive ozone (B, blue) is only moderately increased relative to piControl run A, but there is a clear amplitude increase under 4xCO_2_ without ozone feedbacks (C1, red; also C2, see Table 1 and the supporting information Figure S1). Specifically, *σ*_NINO3.4_ increases from ~0.8 K under preindustrial conditions to ~1.2 K in C1/C2 (~0.9 K in B). This is consistent with the pronounced increase in the number of SST anomalies of magnitudes greater than 1 K in C1/C2, i.e., with more extreme ENSO events. The use of preindustrial (i.e., CO_2_‐level consistent) ozone in the noninteractive piControl simulations A1/A2 leads to no significant difference in ENSO amplitudes relative to A (Table 1 and Figure S1).

Simulations D1/D2 with interactive chemistry from the surface to three model levels above the tropopause capture some part of the ozone response to CO_2_ forcing but are still different to the fully interactive 4xCO_2_ run B (*σ*_NINO3.4_ ~ 1.0 K; see Table 1, Figure S1, and discussion on ozone responses below). D1/D2 are not discussed in detail here (see supporting information) but highlight that this treatment of ozone in climate models can also have a pronounced effect on ENSO responses to CO_2_ forcing.

ENSO amplitudes in other standard index regions provide similar results, see, e.g., the NINO3 (150W–90W, 5N–5S) amplitudes given in Table 1. Crucially, we find not only varying levels of SST variability between the simulations but also a corresponding spread in rainfall rate anomalies. Figure 1b shows precipitation rate anomalies in A, B, and C1 in the NINO3.4 region. There is a general tendency for rainfall variability to increase under 4xCO_2_ with the distributions being skewed toward large positive anomalies. However, the effect is much more prominent (both with respect to anomalously dry and wet periods) in C1/C2 where ozone feedbacks are not included.

### 3.2 The Mechanism

The ENSO amplitude effect can mainly be connected to circulation‐driven ozone decreases in the tropical upper troposphere to lower stratosphere (UTLS) under CO_2_ forcing that are not captured in the noninteractive simulations. The changing ozone impacts the atmospheric lapse rate, which affects the strength of the tropical Walker circulation.

**Figure 2:**
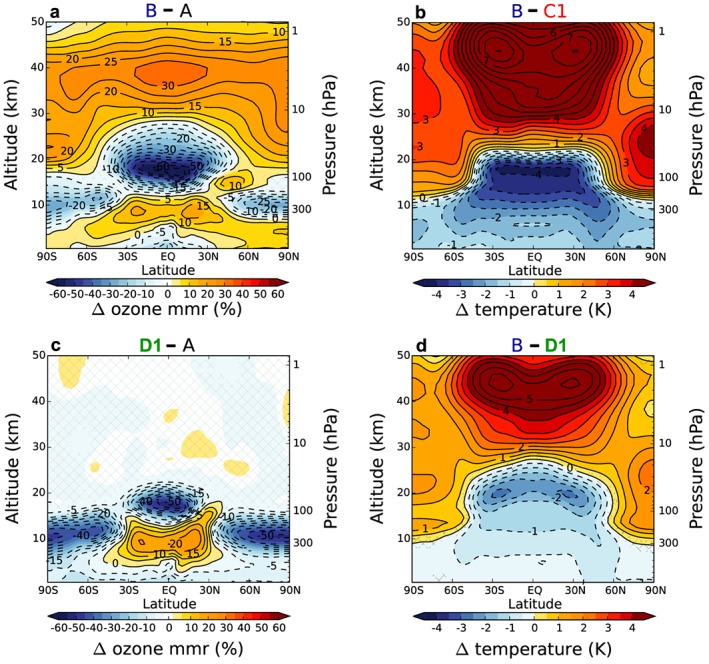
Relative differences (%) in (a and c) zonal mean ozone and (b and d) absolute temperature averaged over 150 years each for the pairs of integrations (as labeled). Nonsignificant changes at the 95% confidence level (using a two‐tailed Student's *t* test) are hatched out. In Figures 2b and 2d, the color scale is constrained to highlight the changes around the tropical tropopause, while the contour lines show the full extent of all changes as 0.5 K intervals. Corresponding differences for C2 and D2 are given in the supporting information Figure S2.

Figure 2 shows latitude‐height cross sections of changes in zonal mean ozone mass mixing ratios and temperatures between the various simulations. There is a decrease in tropical UTLS ozone (Figures 2a and 2c) in 4xCO_2_ runs B and D1 relative to preindustrial conditions (run A and thus also by design C1/C2) within ~30N–30S. Such decreases in tropical UTLS ozone are ubiquitous features in chemistry‐climate model simulations under increased atmospheric greenhouse gas concentrations that have mainly been explained by an acceleration of the stratospheric Brewer‐Dobson circulation [*Lin and Fu*, 2013; *Dietmüller et al*., 2014; *Nowack et al*., 2015]. Middle to upper stratospheric ozone increases found in the fully interactive run B (Figure 2a) under CO_2_‐induced cooling of the stratosphere (Figure S3) are also well understood [*Haigh and Pyle*, 1982; *Jonsson et al*., 2004].

Ozone is a key absorber of *both* solar and terrestrial radiation in the tropical UTLS [*Fueglistaler et al*., 2009; *Riese et al*., 2012; *Ming et al*., 2016]. Therefore, the decreases in ozone have a pronounced cooling effect there (Figures 2b and 2d), with the peak impact located just around the tropical tropopause. However, the changes in tropical UTLS ozone also impact the vertical temperature gradient (i.e., the lapse rate) of the middle to upper tropical troposphere (Figures 2b, 2d, and S4). This is primarily a result of less downwelling long‐wave radiation when tropical UTLS ozone concentrations decline [*Forster et al*., 2007]. Overall, the ozone feedback sharpens the negative temperature gradient with altitude, with the peak lapse rate impact located at the entry to the tropical tropopause layer [*Fueglistaler et al*., 2009] at ~12–14 km altitude (Figure S4).

Simulations D1/D2 include ozone chemistry and the circulation feedback around the tropopause. The method used in D1/D2 thus also avoids a mismatch between the prescribed ozone profile and the atmospheric pressure and temperature profiles in this region, which contributes to the upper tropospheric temperature difference between C1/C2 and B [*Dietmüller et al*., 2014; *Nowack et al*., 2015]. However, the method fails to capture the magnitude and spatial extent of the ozone decreases in the lower stratosphere (compare Figures 2a and 2c). Overall, this gives rise to higher tropical UTLS temperatures in D1/D2 than in B (Figure 2d), albeit cooler than in C1/C2 with fixed preindustrial ozone. In summary, D1/D2 pose an intermediate case with respect to both tropical UTLS ozone and temperature changes. Accordingly, also the subsequent effect on the ENSO lies in between C1/C2 and B (see below). Furthermore, even though the same stratospheric ozone climatologies are used, there are significant middle and upper stratospheric temperature differences between simulations C1/C2 and D1/D2. These temperature differences originate primarily from different levels of stratospheric water vapor changes; comparing C1 and D1, we find a clear stratospheric cooling signature, consistent with greater water vapor increases in C1.

**Figure 3:**
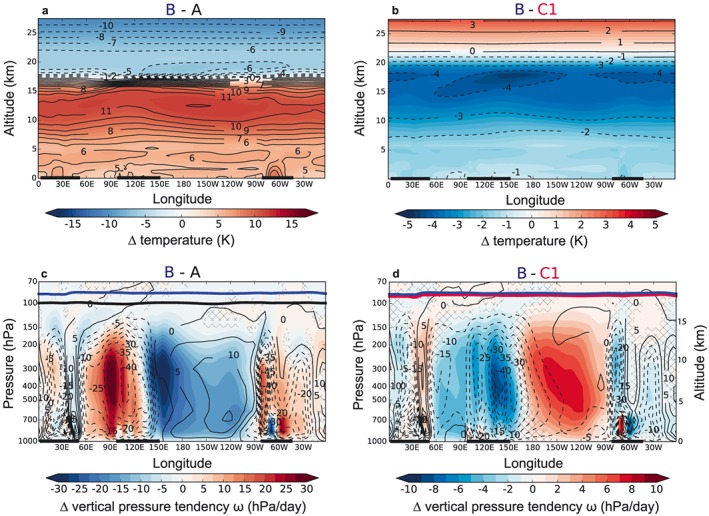
The 5N–5S averages. (a and b) Absolute temperature differences (K). (c and d) Differences in air parcel pressure tendencies *ω* (hPa/d, shaded) and absolute *ω* (contours) from A in Figure 3c and from B in Figure 3d. Annual mean tropopause heights are indicated in corresponding colors. Nonsignificant changes (using a two‐tailed Student's *t* test at the 95% confidence level) are hatched out in Figures 3c and 3d.

To explain the effect of the ozone‐related lapse rate impact, we start with the overall Walker circulation response to CO_2_ forcing and then compare it to the changes induced by the ozone feedback. Figures 3a–3d show longitude‐height cross sections of differences in temperature and vertical pressure tendencies (visualizing circulation changes) averaged over 5N to 5S. Figures 3a and 3c show the difference between simulations A and B, i.e., the full effect of all changes, while Figures 3b and 3d isolate the ozone impact (B minus C1).

In agreement with the majority of state‐of‐the‐art models, we find an on average weakened Walker circulation in response to the CO_2_ forcing [*Vecchi et al*., 2006; *Vecchi and Soden*, 2007; *Collins et al*., 2010; *Christensen et al*., 2013; *Bayr et al*., 2014]. This is characterized by decreased upwelling velocities above the Maritime Continent and West Pacific (WP) and smaller net downward velocities above the Central Pacific and East Pacific (EP) (Figure 3c). A key factor behind this robust slowing down of the Walker circulation is that the middle to upper troposphere has typically been found (Figure 3a) to warm more than the lower troposphere under CO_2_ forcing [*Ma et al*., 2012; *Christensen et al*., 2013; *Bayr et al*., 2014]. The tropospheric lapse rate is thus reduced under CO_2_ forcing in climate models. This implies slower tropical convective mass fluxes [*Held and Soden*, 2006], which is equivalent to a weakened Walker circulation [*Vecchi and Soden*, 2007; *Bayr et al*., 2014].

The characteristic temperature impact of the changes in tropical UTLS ozone (Figure 3b) opposes this CO_2_‐induced lapse rate feedback (Figures 3a and S4/S5). Accordingly, it also changes the conditions for tropical deep convection and the Walker circulation. Including ozone feedbacks gives rise to a relative destabilization of the tropical middle to upper troposphere (Figure S6), consistent with strengthened deep convection and collocated deep convective precipitation above the tropical WP and Maritime Continent (Figure S7). As a result, ozone feedbacks damp the slowing of the Walker circulation under CO_2_ forcing.

On average, the Walker circulation maintains easterly surface winds across the tropical Pacific. A consequence of the zonal wind stress is upwelling of cold, deep ocean waters in the EP. Combined with the cold northward Humboldt current along the South American coastline, the oceanic upwelling gives rise to the formation of unusually low SSTs in the EP [*Vecchi and Wittenberg*, 2010]. The weaker Walker circulation in all 4xCO_2_ simulations leads to significantly reduced average zonal surface wind stress (Figure S8). Accordingly, EP oceanic upwelling is much reduced in each 4xCO_2_ run. However, the reduction is almost twice as large in those cases without ozone feedbacks, i.e., C1/C2 (Figure S9a), consistent with the enhanced weakening of the Walker circulation in these cases. The oceanic and wind stress differences between the simulations further lead to a reorganization of the upper ocean heat budgets (Figures S9b–S9e).

**Figure 4:**
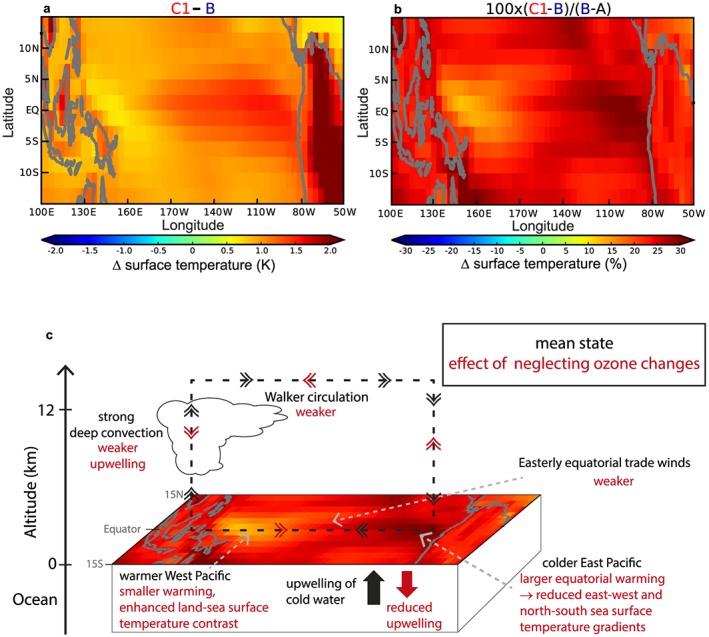
(a) Surface temperature differences (K) between the 4xCO_2_ runs with preindustrial ozone (C1) and interactive chemistry (B) for the region 15N–15S, 100E–50W. (b) The same temperature anomalies expressed as percentage differences relative to the surface warming caused by 4xCO_2_ from run A to B. (c) Sketch summarizing the climatological mean state effect of neglecting ozone changes.

The mean state differences in wind stress and EP oceanic upwelling of cold water have a characteristic effect on tropical Pacific SSTs (Figures 4a, 4b, and S10). In particular, the additional surface warming in C1/C2 due to missing ozone feedbacks:

1. is greater in the EP than in the WP, resulting in an on average reduced zonal SST gradient; the SST gradient between the NINO4 (WP, 160E–150W, 5N–5S) and NINO3 (EP) regions is reduced in C1/C2 relative to A (by approximately −0.05 K), whereas it is increased in B (by approximately 0.25 K);
2. is greater on than off the equator in the EP, which reduces the EP meridional SST gradient; and
3. is smaller in the WP than on the neighboring Maritime Continent (Figures 4a and 4b).

The spatial structure of the SST differences between C1/C2 (or D1/D2) and B is highly consistent, underlining the robustness of the overall ozone‐induced feedback on the tropical Pacific mean state (summarized in Figure 4c).

Such SST differences have been directly related to increases in the number of (extreme) ENSO events in general [*Cai et al*., 2014, 2015a, 2015b; *Kim et al*., 2014], defined by either anomalous SSTs or rainfall (Figure 1). The enhanced EP warming when the ozone feedback is neglected facilitates the formation of strong El Niño events. Larger amplitude El Niño events, in turn, induce more extreme La Niña events, an effect that is further supported by the larger WP to Maritime Continent surface temperature contrast. Based on these previous multimodel results, we argue that these mean state changes are an important driver of the varying ENSO amplitude responses in our 4xCO_2_ simulations.

## 4 Conclusions

The response of the ENSO to climate change is still highly uncertain [*Christensen et al*., 2013; *Bellenger et al*., 2014; *Cai et al*., 2015b; *Ham and Kug*, 2016; *Rashid et al*., 2016]. Here we demonstrate how model representations of ozone can impact ENSO amplitude projections. The effect of CO_2_ forcing is to reduce the vertical temperature gradient of the tropical troposphere in climate models, which acts to slow down the Walker circulation. CO_2_‐driven changes in tropical UTLS ozone counteract this effect in our model. The Walker circulation is intrinsically coupled to the ENSO so that the changes in ozone eventually lead to adaptations in the tropical Pacific mean state (SST gradients and land‐sea temperature contrasts) that tend to reduce amplitudes of ENSO events.

It is well known that the modeled magnitude of upper tropospheric warming under greenhouse gas forcing is uncertain [*Fueglistaler* *et al*., 2015; *Sohn* *et al.*, 2016]. In addition, models differ in their zonal mean and regional ENSO changes under climate change [*Christensen et al*., 2013; *Bellenger et al*., 2014]. However, circulation‐driven decreases in tropical UTLS ozone are a robust feature in chemistry‐climate models without an interactive ocean [*SPARC*, 2010]. Therefore, this part of the ozone response is largely decoupled from the ENSO response itself, including the underlying structure of SST changes and tropospheric warming. As a result, it seems reasonable to assume that similar lapse rate changes should occur in other climate models once ozone feedbacks are included, although their relative importance might differ. Model sensitivities in developing an initial ozone anomaly and triggering a lapse rate adjustment are highly variable. Our model has a high sensitivity, whereas *Marsh et al.* [2016] reported a negligible impact. A study by *Dietmüller et al.* [2014] (using a mixed layer ocean) showed a similar behavior to our model, but with a lower magnitude. We note that the zonal mean and regional changes (Figure 3) are closely coupled; however, no detectable change in the zonal mean might not necessarily equate to no regional changes. Therefore, we highlight the regional changes of the Walker circulation in response to the lapse rate adjustment. Interestingly, *Chiodo and Polvani* [2015] have traced the impact of solar cycle‐induced ozone changes in idealized model experiments onto the strength of the Walker circulation, thus highlighting the importance of a zonal asymmetric response (weakening of the Walker circulation) to a largely zonal symmetric forcing (ozone anomaly), as we find it in our model under global warming.

In conclusion, we highlight the coupling between tropical ozone, circulation, and precipitation changes as an important matter to address for the scientific community. For this, we see a crucial need to test the intermodel robustness of the ENSO effect described here and other chemistry‐climate feedbacks in a range of ocean‐coupled chemistry‐climate models.

## Supporting information



Supporting Information S1
