# Supplementary material for: On the role of ozone feedback in the ENSO amplitude response under global warming
Source: Geophys Res Lett. 2017 Apr 30;44(8):3858–66. doi: 10.1002/2016GL072418 (PMC5518766; doi:10.1002/2016GL072418)
Supplement: Supplementary file 1 — Supporting Information S1 [file GRL-44-3858-s001.pdf]

AGU PUBLICATIONS

Geophysical Research Letters

Supporting Information for

On the role of ozone feedback in the ENSO amplitude response under global warming

Peer J. Nowack[1*], Peter Braesicke[2], N. Luke Abraham[1,3] and John A. Pyle[1,3]

[1]Department of Chemistry, Centre for Atmospheric Science, University of Cambridge, Cambridge, United Kingdom

[2] Karlsruhe Institute of Technology, IMK-ASF, Karlsruhe, Germany

[3] National Centre for Atmospheric Science, United Kingdom

Contents of this file

Text S1: Further details on the simulations
Text S2: Further details on the statistical analysis
Figures S1 to S11

Introduction

This file contains additional explanations on the simulations and the statistical analysis followed by several figures to support arguments made in the main manuscript.

Text S1: Further details on the simulations

In the main text, we mention two runs for each 'non-interactive' case, in which ozone and other major radiatively active trace gas species (methane, nitrous oxide) are imposed as time-averaged monthly-mean fields that cover both the seasonal cycle and the model's spatial dimensions. In the first case, these climatologies were imposed as a full 3D (latitude, longitude, height) fields of ozone mass mixing ratios (label 1). Alternatively, these climatologies were additionally longitudinally averaged and as such imposed as a 3D field, see also *Nowack et al.* [2015]. The chemical climatologies are based on monthly-mean values, but are updated every five days via linear interpolation. The two types of simulations produce very similar results, i.e. differences between these two versions of non-interactive simulations are small compared to the larger differences in ozone caused by a 4xCO_2 increase. As a result, we consider simulations C1/C2 (as well as D1/D2) effectively as two ensemble members of the same type of experiment in our study.

Text S2: Further details on the statistical analysis

S2.1 Data processing

As mentioned in the main text, years 50-200 of each 200-year long 4xCO_2 and 150 years of each piControl simulation were used for statistical analysis. The data used for calculating ENSO indices is linearly de-trended to account for remaining surface temperature trends in the abrupt 4xCO_2 simulations. The pre-industrial experiments are equally de-trended, however, without noticeable effect. A comparative analysis of ENSO indices under 4xCO_2 before and after the de-trending showed that the de-trending does not affect the conclusions. It is nevertheless carried out for the sake of consistency. Where needed, the data was linearly interpolated to a 1°x1° horizontal grid to obtain specific grid coordinates (e.g. for the SST-based ENSO indices).

S2.2 Statistical significance testing

The differences between simulations that only differ by the use of either zonal mean or full 3D chemical climatologies, i.e. C1/C2, A1/A2, or D1/D2 are small. Therefore, the sets of 2D and 3D climatology runs are considered as approximately two ensemble members. In addition, two shorter non-interactive 4xCO_2 runs of 75 years length with ozone imposed from interactive run B (hereafter referred to as B1/B2) were carried out [*Nowack et al.*, 2015], with essentially identical results to the fully interactive case, which further supports the robustness of the results. Taken together, the consistency in ENSO amplitudes between similar simulations (C1/C2, D1/D2, B/B1/B2, A/A1/A2) of in total 1300 years, where each is started from different initial piControl conditions, underlines the significance of the differences in ENSO amplitudes between the sets of experiments given the long data records needed to analyze details of ENSO changes implied by earlier studies [*Wittenberg*, 2009; *Stevenson et al.*, 2010]. Additionally, 300 years of data from pre-industrial run A were tested on their inter-centennial variation by calculating ENSO amplitudes for forward moving windows of 150 years, with the windows shifted by 10 years at a time. This provided standard deviations ranging from 0.72 to 0.82 K for the NINO3.4 region and 0.68-0.75 K for the NINO3 region. Overall, this confirms that the ENSO is significantly different between the sets of simulations (Table 1), in particular between C1/C2 and B, above the level of natural long-term variability within the model. Furthermore, the differences do not only occur after long-time averaging over 150 years. For instance, NINO3.4 and NINO3 amplitudes were calculated using 50-year windows of monthly-mean SST anomalies (Fig. S11). There is variability between different 50-year windows, however, the greater amplitudes in C1/C2 than in B and A/A1/A2 occur throughout the whole 150 years considered for each simulation.

Supplementary Figures

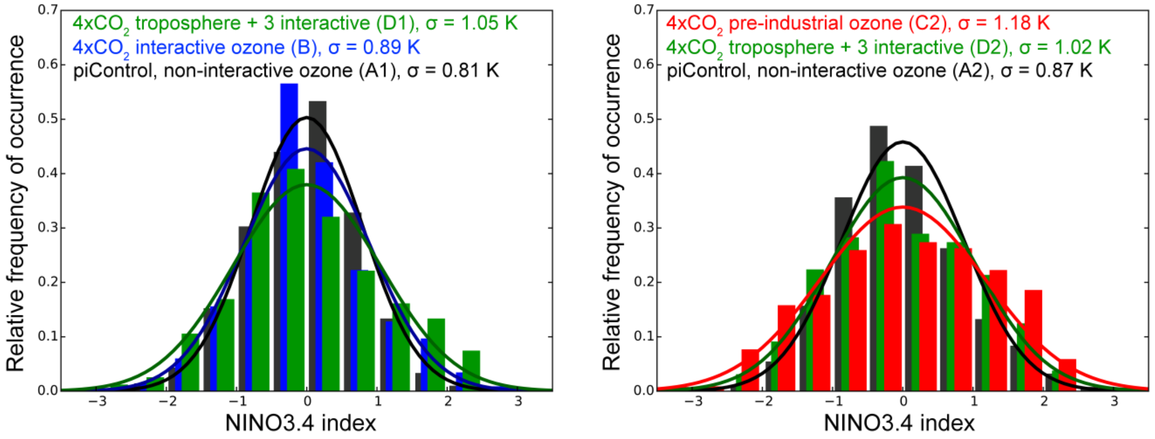

Figure S1. Additional NINO3.4 histograms. As for Figure 1a in the main text, but now for the two non-interactive pre-industrial control runs A1 and A2 as well as simulations C2 and D1/D2. The model set-up for each of the simulations is described in the main text.

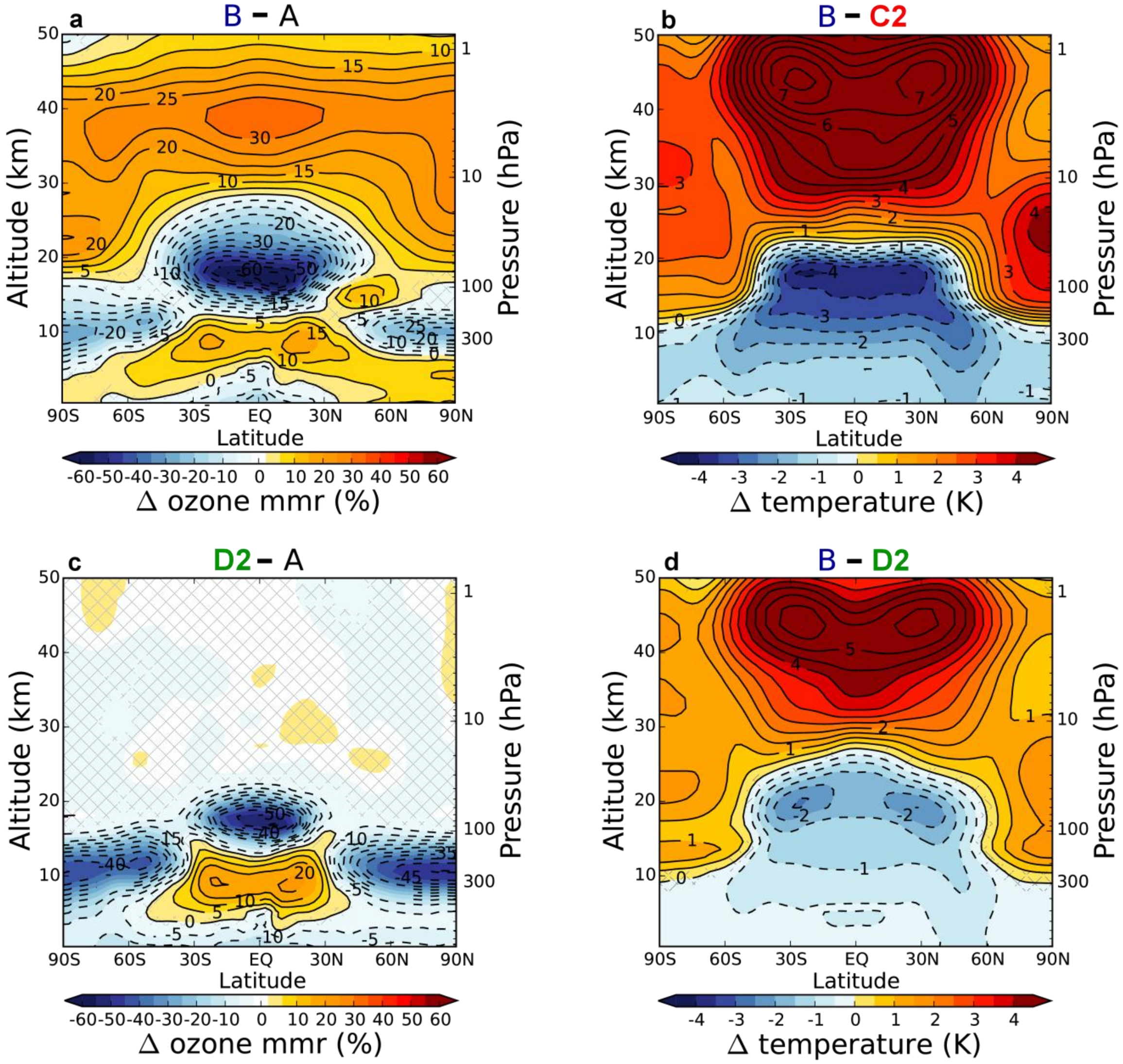

Figure S2. Zonal mean ozone and temperature changes. As for Figure 2 in the main text, but with D1 replaced by D2 and C1 by C2. Small artifacts in the non-interactive region in (c) result from ozone changes between the dynamical and chemistry model time-steps, which occur before the ozone field is reset to pre-industrial values. The specification of constant ozone mass mixing ratios from three model levels above the tropopause limits ozone decreases in the lower stratosphere. Neglecting the temperature-driven middle-to-upper stratospheric ozone increases also leads to relatively higher ozone production rates in the lower stratosphere [*Haigh and Pyle*, 1982; *Nowack et al.*, 2016]. As a result, the decreases in tropical UTLS ozone in D1/D2 are smaller than in the fully interactive case B, which explains the smaller cooling of the tropical UTLS due to the changes in ozone, see sub-figure (d). Note that temperature differences in the stratosphere between the simulations are not only driven by changes in ozone. In particular differences in stratospheric water vapor play a key role [Nowack et al., 2015]. Specifically, ozone feedbacks reduce stratospheric water vapor increases by ~4-5 ppmv in B and ~2-3 ppmv in D1/D2 (relative to C1/C2).

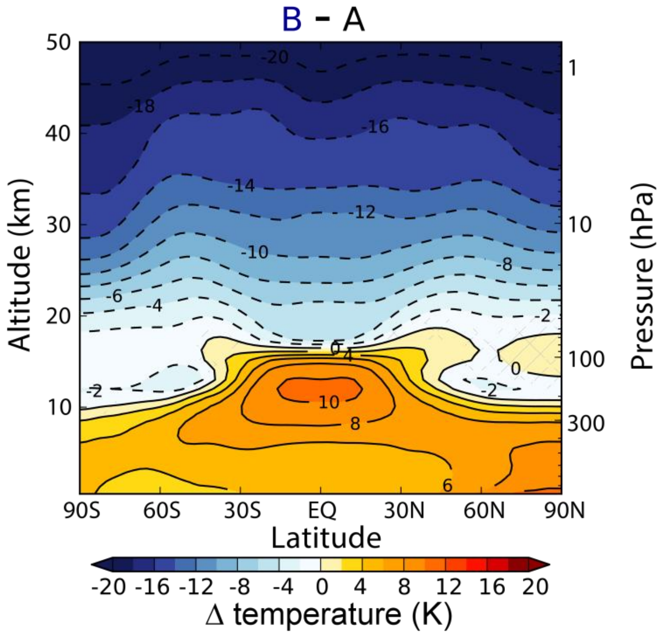

Figure S3. Zonal mean temperature changes. Here between B and A, for years 25-75 after the 4xCO_2 forcing in B. The figure shows the tropospheric warming and stratospheric cooling under increased atmospheric carbon dioxide levels.

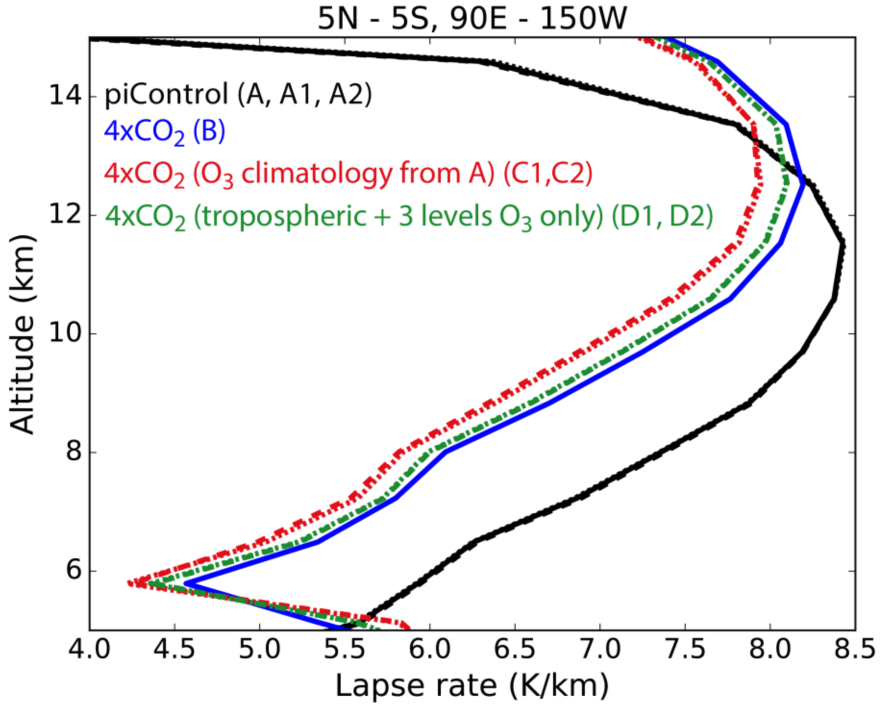

Figure S4. Climatological lapse rates (-dT/dz). Shown are climatological vertical temperature gradients for the various piControl and 4xCO_2 runs in the area of deep reaching convection within 90E to 150W, 5N to 5S. Dashed (dotted) lines represent runs with 3D (2D) chemical climatologies. The low-level lapse rate minimum (in terms of the absolute value) is shifted upwards in the 4xCO_2 simulations and is therefore not included for A/A1/A2 (for which this point occurs at ca. 4.5 km). Interactive runs are marked by solid lines. Colors as labeled.

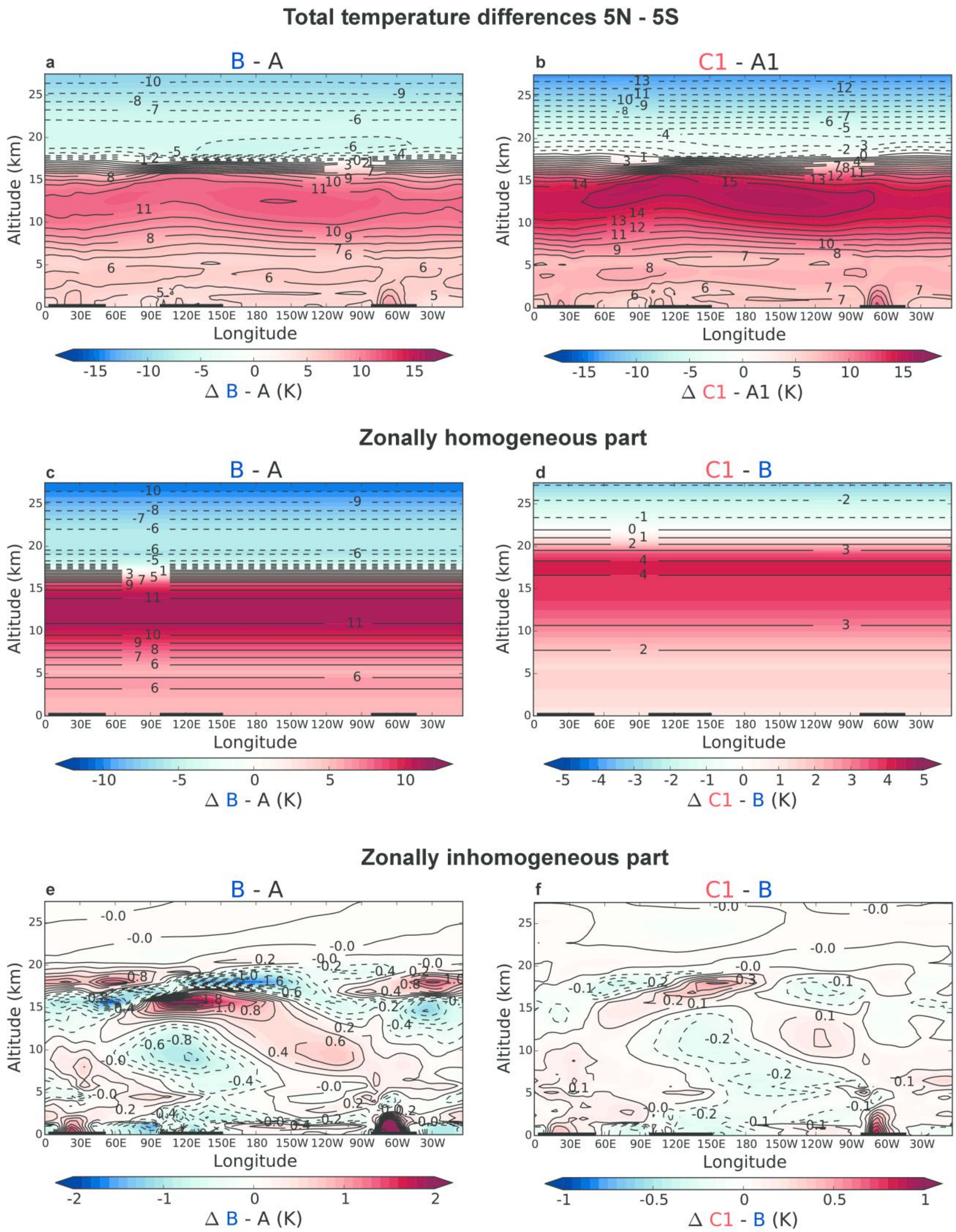

Figure S5. Total, zonally homogeneous and inhomogeneous annual mean warming of the tropical atmosphere between 0 and 25 km, within 5N to 5S. Differences as labeled, with homogeneous meaning the zonal mean and inhomogeneous zonal anomalies with respect to the zonal mean, following Bayr *et al.* [2014]. The differences highlight the significant and characteristic imprint of the tropical UTLS ozone changes on the vertical temperature structure. Changes in the vertical temperature gradient are key to the strength of convection and thus for the Walker circulation. Black lines at the ground mark land areas, where strong convection occurs. Continuation see next page.

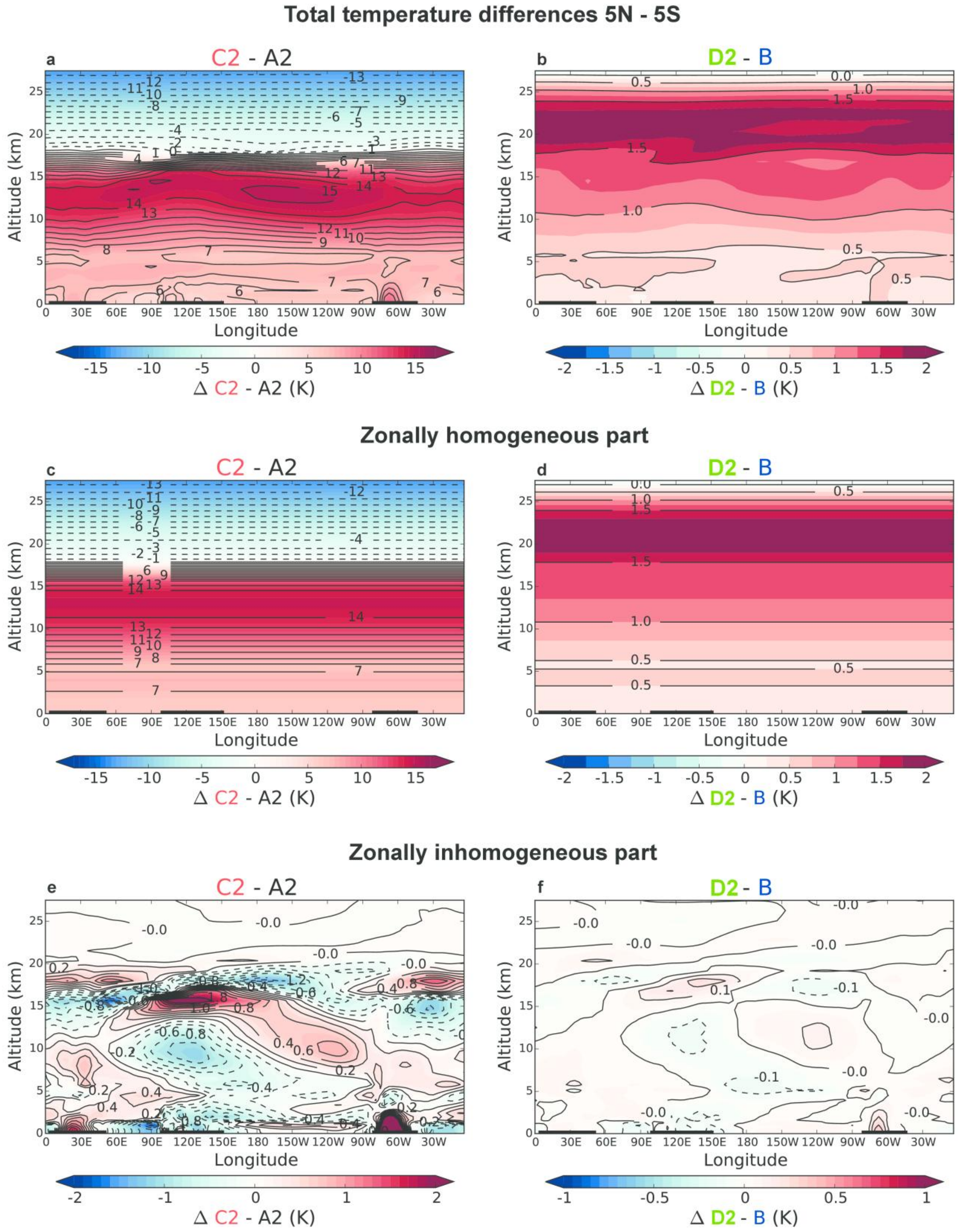

Figure S5 continued. Total, zonally homogeneous and inhomogeneous annual mean warming of the tropical atmosphere between 0 and 25 km, within 5N to 5S. Now for C2 and D2 as labeled. Note that the tropical UTLS ozone feedback's temperature impact is fairly zonally homogeneous, implying that changes in the local heating rates of the WP and EP are secondary, just as for the hydrological cycle feedback mechanism. Indeed, the inhomogeneous warming differences above the tropical Pacific can be connected to the different Walker circulation and thus SST responses in the runs (Figures 3,4 and S10). Note the different color scales in the various sub-plots to highlight structures of the individual temperature changes.

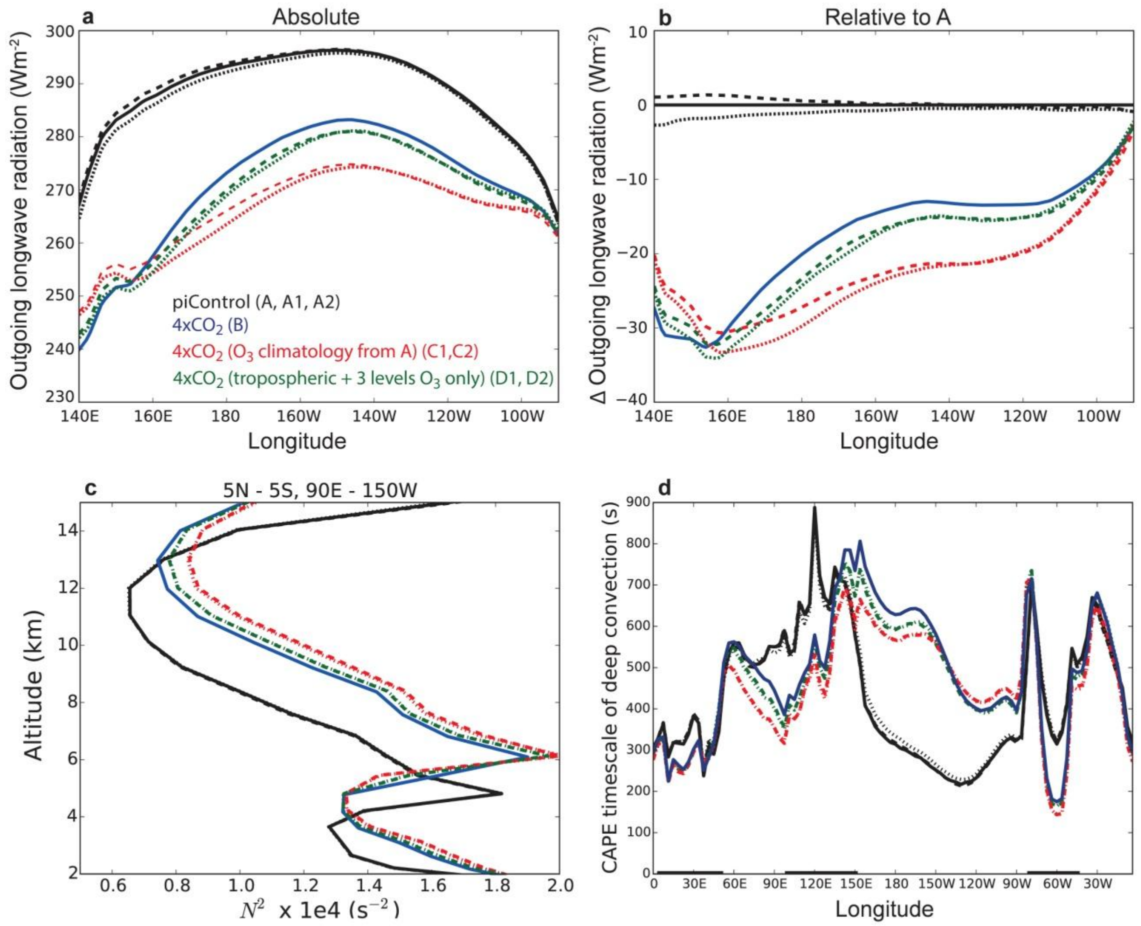

Figure S6. Several diagnostics to demonstrate the impact of the lapse rate changes on convection in 5N to 5S. Dashed (dotted) lines mark runs with 3D (2D) chemical climatologies, solid lines denote interactive runs. Descriptions below.

Description of Figure S6:

- (a) Outgoing long-wave radiation (OLR, Wm^{-2}). Changes in convection are often diagnosed using the OLR at the top of the modeled atmosphere. The OLR is well correlated with the strength of convection in regions of dense cloud cover, where it is primarily being emitted from the highest convective cloud tops, with high reaching convective cloud tops implying strong convection. Since higher reaching clouds emit at lower temperatures, they emit less energy to space. Consequently, increased/higher-reaching convection is expected to cause OLR decreases and vice versa. In all $4xCO_2$ simulations, total OLR decreases relative to pre-industrial conditions, i.e. with increasing surface temperatures. However, the decrease is disproportionally large in the CP and EP in C1/C2 relative to B and D1/D2. This is consistent with the also disproportionally higher surface temperatures there (Figure 4 in the main text and Figure S10), which give rise to increased convection in this mean downwelling region. In contrast, there is *more* OLR in C1/C2 than in D1/D2 and B above the highly convective upwelling region above the WP (ca. 140E-160E), in spite of the higher surface temperatures in C1/C2. So, the differences in OLR in this region between C1/C2 and B/D1/D2 oppose the trend expected from the differences in surface temperature. This demonstrates the specific role of ozone in modulating convection.
- (b) The same as (a), but relative to pre-industrial run A.

- (c) Brunt-Vaisälä frequencies N^2 (in s^{-2}) averaged over 90E to 150W. N^2 effectively gives the buoyancy frequency of an air parcel that is vertically displaced from a stable position within the atmosphere. Negative N^2 implies unstable conditions under which convection occurs. The annual mean atmosphere (here calculated from monthly-mean data of potential temperature) is stable, but N^2 is most positive in C1/C2. This implies less convective potential in C1/C2 than in B, in agreement with the destabilizing lapse rate impact of the ozone feedback (Figure S4).
- (d) Convective available potential energy time-scale of deep convection. Longer deep convective time-scales imply greater convective strength and higher penetration of convection, i.e. a less stable atmosphere. In all $4xCO_2$ runs the convective centre above the Maritime Continent is shifted eastward and the maximum CAPE time-scale decreases. Next to the general stabilization of the atmosphere due to the homogeneous warming mechanism, this implies an eastward shift of the Walker circulation cell. Such a shift is indeed identified in other atmospheric flow diagnostics (Figure 3 in the main text). Deep convective strength is greater in B than in C1/C2 within this convective centre, in spite of the lower surface temperatures. That this effect is particularly large over the Maritime Continent and over the WP is likely mainly caused by the particularly high reaching deep convection in this area (reflected in the longest time-scales). Since the lapse rate impact of the ozone feedback maximizes in the upper troposphere, this region is necessarily more affected than other regions where convection does not often penetrate as deep. The plots are based on average data over years 50-200 of each $4xCO_2$ simulation and over 150 years of each pre-industrial run.

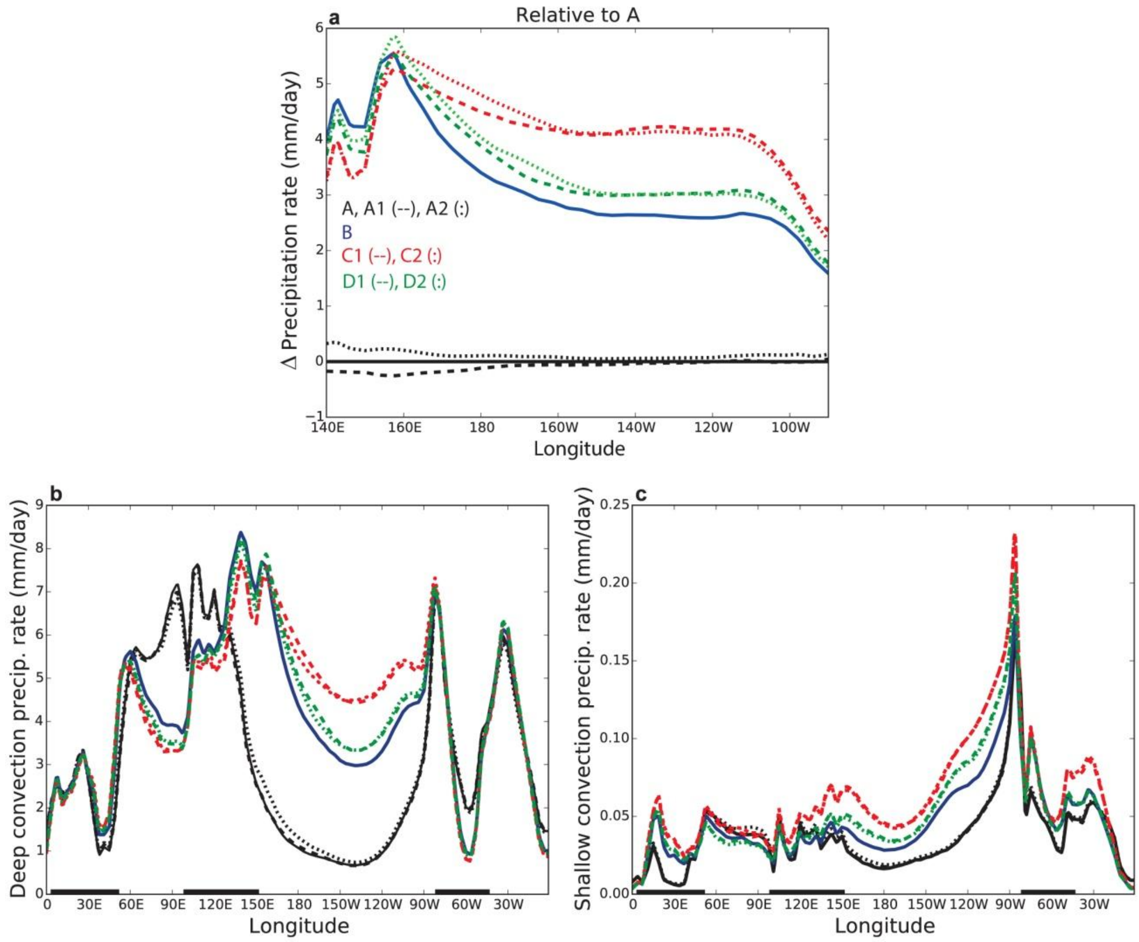

Figure S7. Precipitation rate changes within 5N-5S (mm/day). As labeled. (a) Total annual mean precipitation rate changes relative to the pre-industrial control experiment, A, above the Pacific. The differences are based on years 50-200 of each 4xCO_2 simulation and 150 years of each piControl run. There is a clear relative increase in precipitation in the CP and EP in C1/C2 as compared to D1/D2 and B. The particularly large rainfall increase in C1/C2 in the CP and EP can be explained by the larger SST increase there than in B/D1/D2, with the warming also being stronger on the equator than off the equator (Figures 4 and S10), which attracts rainfall. At the same time, there is less precipitation in C1/C2 in the WP, in contrast to the expected trend purely by changes in surface temperature. This implies the specific role of the ozone feedback in modulating convective rainfall in this highly convective region. The results further underline that D1/D2 pose an intermediate case between C1/C2 and B. (b) Absolute deep convective rainfall rates, which dominate the overall rainfall in the inner tropics. Precipitation rate differences between C1/C2 and B are particularly large above the Pacific and of opposite sign between the WP and CP/EP. This consolidates deep convective changes as the key driver behind the overall differences in precipitation rates. As shown in (c), there are also significant differences in shallow convective precipitation. However, compared to the deep convective precipitation changes they are of small magnitude and are well correlated with changes in surface temperature rather than with specific ozone impacts.

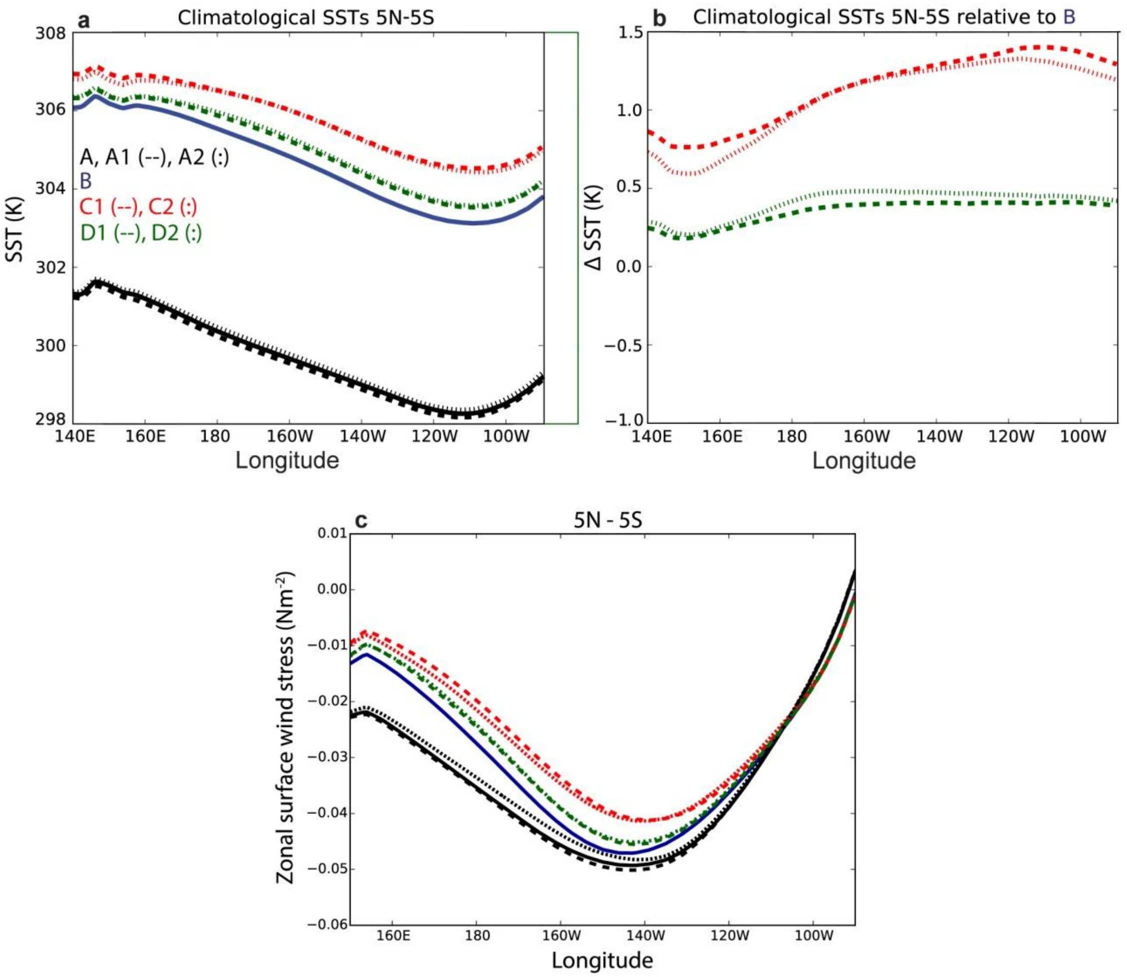

Figure S8. Climatological sea surface temperatures and zonal surface wind stress in the tropical Pacific within 5N to 5S. As labeled. Data obtained from years 50-200 of each abrupt 4xCO_2 simulation and 150 years of each piControl run is used to calculate the averages. (a) Absolute SSTs, (b) C1/C2 and D1/D2 relative to B, highlighting the greater EP than WP warming in C1/C2 in particular. (c) Surface zonal wind stresses for the various runs. Wind stresses are much reduced in the CP and WP in C1/C2, in agreement with the weaker Walker circulation and smaller zonal SST gradient in these runs.

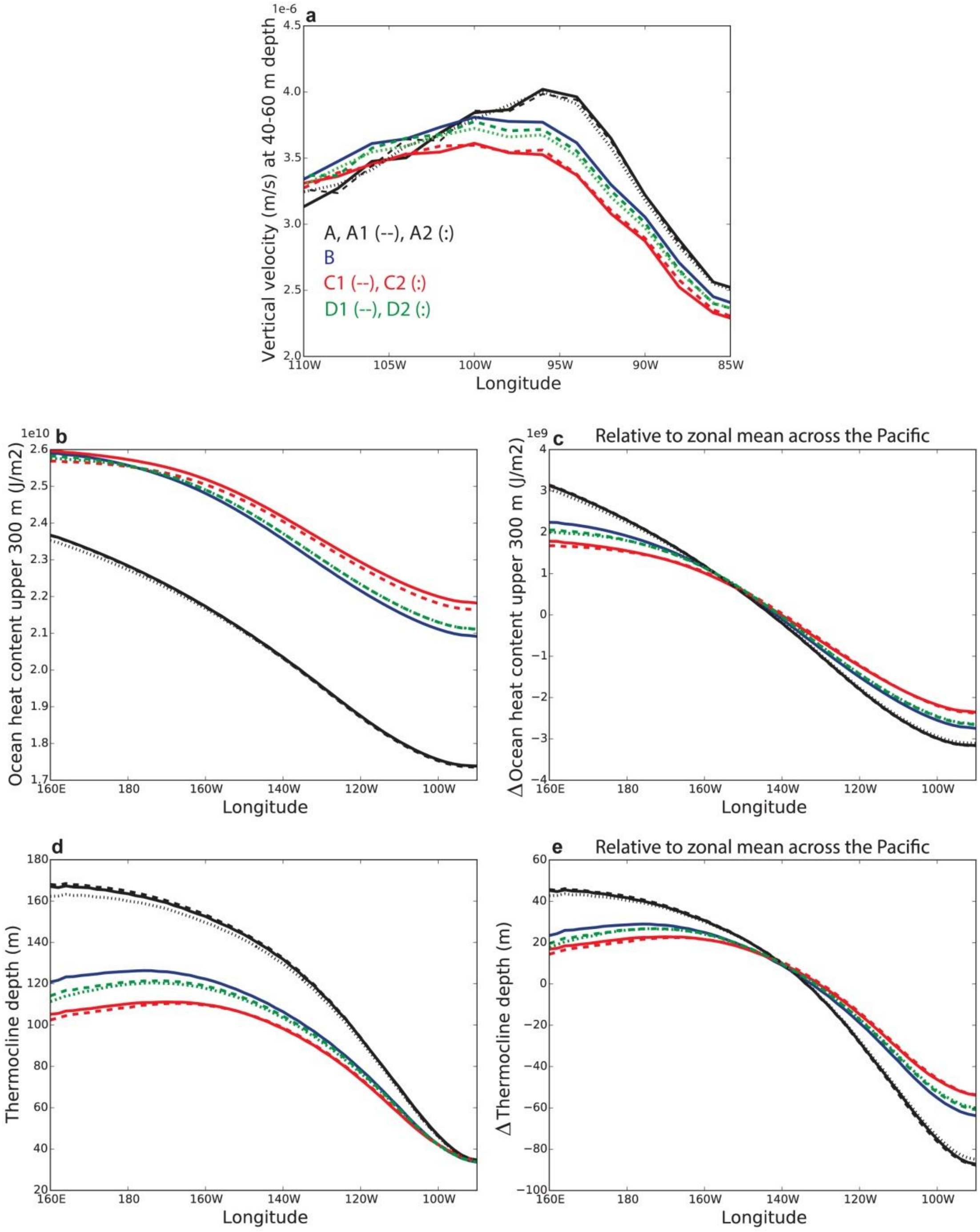

Figure S9. Annual mean changes in oceanic upwelling velocities, upper ocean heat content and thermocline depth in the Pacific within 5N to 5S. Colors and lines of runs as labeled. (a) Vertical velocities (m/s) averaged over 40-60 m depth in the EP. (b) Ocean heat contents (Jm^{-2}) of the upper 300 m. (c), same as (b) but relative to the zonal mean of each run across the Pacific to demonstrate the zonal redistribution of heat within the Pacific, which is partly driven by the surface winds. (d) Thermocline depths (m). (e), as (d) but relative to the zonal mean of each run, highlighting the zonal flattening of the thermocline under greenhouse gas forcing that is further enhanced as a result of even less EP upwelling and the reduced zonal wind stress in C1/C2 compared to B. Here, the thermocline is defined by the position of the maximal vertical temperature gradient.

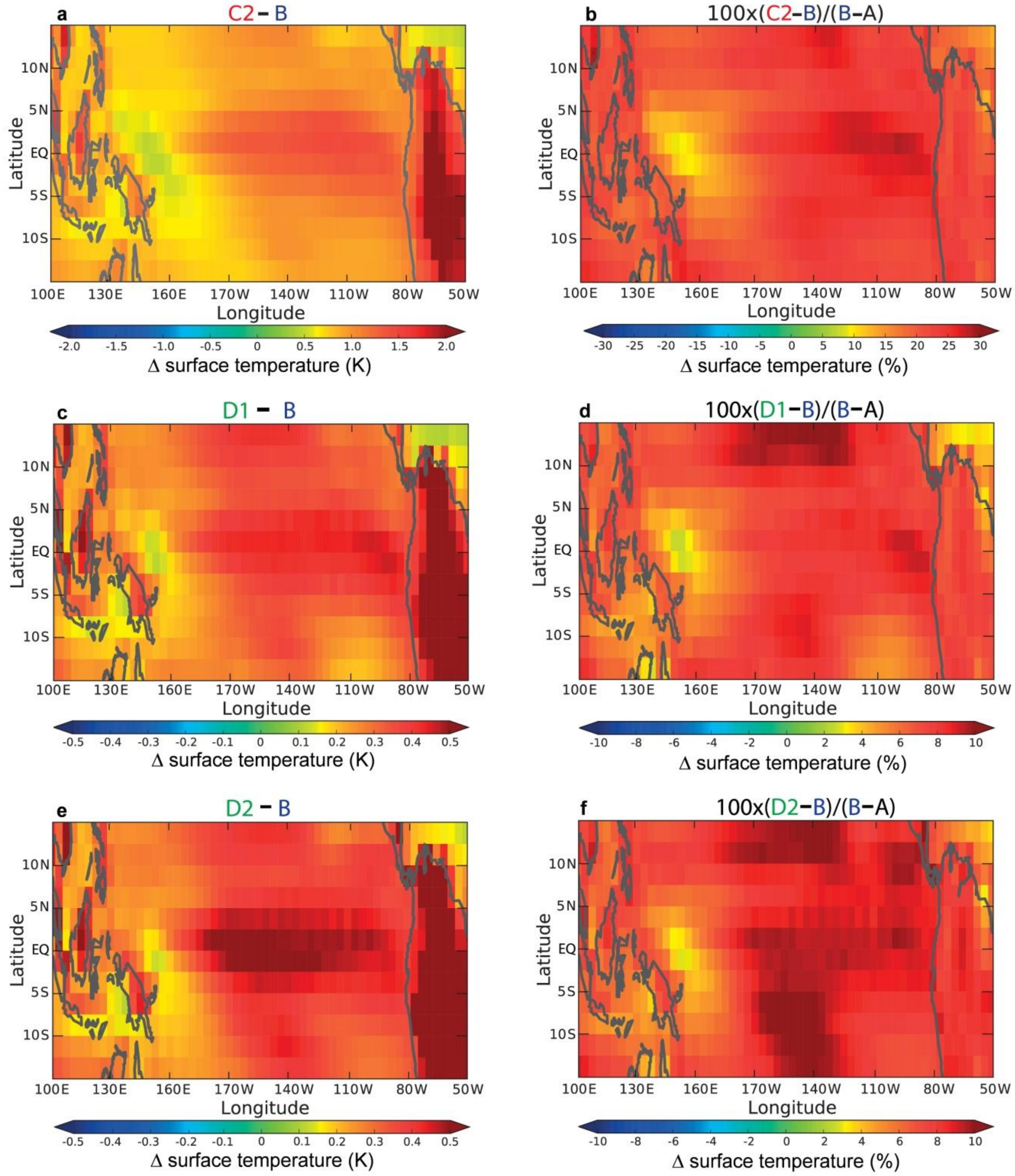

Figure S10. Surface temperature differences. Extension of Figure 4 in the main text, showing surface temperature differences between C2/D1/D2 and B both as absolute values (K, left column) and as percentages relative to the warming from A to B (right column). Even though different in magnitude, the structure of the changes is always similar, with enhanced EP to CP warming and relatively less warming in the WP. Note the different color scales used.

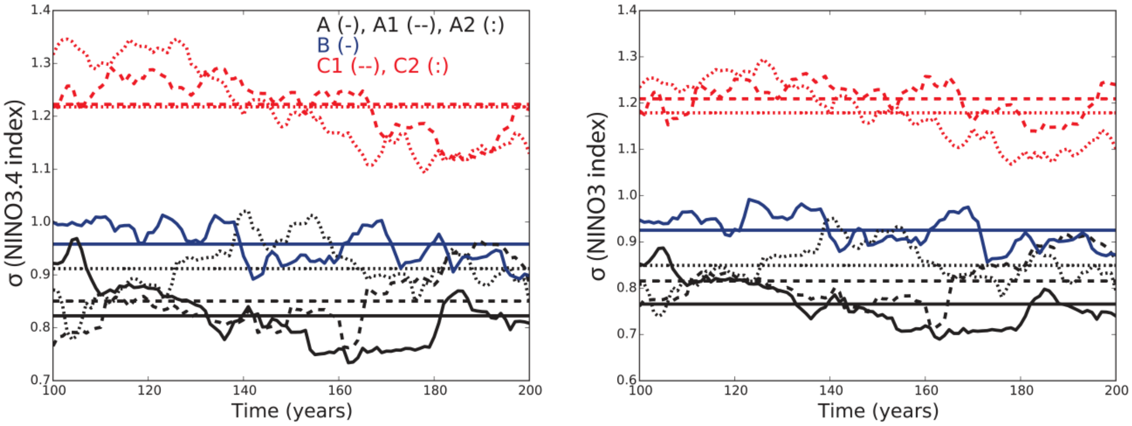

Figure S11. 50-year windows of NINO3.4 and NINO3 index standard deviations σ. As labeled, using de-trended monthly-mean data. Each data point represents the standard deviation of the SST anomalies in the present and the previous 49 years. The first 50 years of each run are not taken into consideration. Hence, the time-line starts in year 100. The straight lines mark the average σ over all 50-year windows (not identical with the σ calculated for the whole 150 years from 5-month running mean data in Figure 1a and Table 1 in the main text.
